# Deregressing estimated breeding values and weighting information for genomic regression analyses

**DOI:** 10.1186/1297-9686-41-55

**Published:** 2009-12-31

**Authors:** Dorian J Garrick, Jeremy F Taylor, Rohan L Fernando

**Affiliations:** 1 Department of Animal Science, Iowa State University, Ames, IA 50011, USA; 2 Institute of Veterinary, Animal & Biomedical Sciences, Massey University, Palmerston North, New Zealand; 3 Division of Animal Sciences, University of Missouri, Columbia 65201, USA

## Abstract

**Background:**

Genomic prediction of breeding values involves a so-called training analysis that predicts the influence of small genomic regions by regression of observed information on marker genotypes for a given population of individuals. Available observations may take the form of individual phenotypes, repeated observations, records on close family members such as progeny, estimated breeding values (EBV) or their deregressed counterparts from genetic evaluations. The literature indicates that researchers are inconsistent in their approach to using EBV or deregressed data, and as to using the appropriate methods for weighting some data sources to account for heterogeneous variance.

**Methods:**

A logical approach to using information for genomic prediction is introduced, which demonstrates the appropriate weights for analyzing observations with heterogeneous variance and explains the need for and the manner in which EBV should have parent average effects removed, be deregressed and weighted.

**Results:**

An appropriate deregression for genomic regression analyses is *EBV/r*^2 ^where EBV excludes parent information and *r*^2 ^is the reliability of that EBV. The appropriate weights for deregressed breeding values are neither the reliability nor the prediction error variance, two alternatives that have been used in published studies, but the ratio (1 - *h*^2^)/[(*c *+ (1 - *r*^2^)/*r*^2^)*h*^2^] where *c *> 0 is the fraction of genetic variance not explained by markers.

**Conclusions:**

Phenotypic information on some individuals and deregressed data on others can be combined in genomic analyses using appropriate weighting.

## Background

Genomic prediction [1] involves the use of marker genotypes to predict the genetic merit of animals in a target population based on estimates of regression of performance on high-density marker genotypes in a training population. Training populations might involve genotyped animals with alternative types of information including single or repeated measures of individual phenotypic performance, information on progeny, estimated breeding values (EBV) from genetic evaluations, or a pooled mixture of more than one of these information sources. In pooling information of different types, it is desirable to avoid any bias introduced by pooling and to account for heterogeneous variance so that the best use is made of available information.

Uncertainty as to whether or not EBV should be used directly or deregressed or replaced by measures such as daughter yield deviation (DYD) [2], and the manner in which information should be weighted, if at all, has been apparent for some time in literature related to discovering and fine-mapping quantitative trait loci (QTL). Typically in fixed effects models with uncorrelated residuals, observations would be weighted by the inverse of their variances. Morsci et al. [3] pointed out the counter intuitive behavior of using the reciprocal of the variance of breeding values as weights in characterization of QTL and followed the arguments of Rodriguez-Zas et al. [4] in using reliability as weights. Rodriguez-Zas et al. [4] did analyses that were limited by features of the chosen software so EBV/2 (i.e. predicted transmitting ability PTA) were multiplied by the square root of reliability and analyzed unweighted. Georges et al. [5] deregressed PTA to construct DYD and weighted these using the inverse of the variance of the DYD. Spelman et al. [6] had direct access to DYD and similarly weighted these by the inverse of their scaled variance, equivalent to using the inverse of reliability as weights. Other researchers have reported the use of PTA [7], standardized PTA [7,8] or DYD weighted by respective reliabilities [8]. The uncertainty associated with using information for QTL discovery has recently been extended to genomic prediction. An Interbull survey [9] of methods being used in various countries for genomic prediction of dairy cattle reported that some researchers used deregressed proofs weighted with corresponding reliabilities, others used DYD weighted by effective daughter contributions, while yet others used EBV without any weighting. The objective of this paper is to present a logical argument for using deregressed information, appropriately weighted for analysis. For simplicity, we consider the residual variance from the perspective of an additive model but the deregression and weighting concepts extend to analyses that include dominance and epistasis.

## Methods

### An ideal model

Genomic prediction involves the use of genotypes or haplotypes to predict genetic merit. Conceptually, it involves two phases, a training phase where the genotypic or haplotypic effects are estimated, typically as random effects, in a mixed model scenario, followed by an application phase where the genomic merit of selection candidates is predicted from the knowledge on their genotypes and previously estimated effects from the training phase. The ideal data for training would be true genetic merit data observed on unrelated animals in the absence of selection. In that case, the model equation would be:(1)

where **g **is a vector of true genetic merit (i.e. breeding value BV) with *var*(**g**) = **T**, the scalar  is the genetic variance and **T **can be constructed using the theory from combined linkage disequilibrium and linkage analyses [10], *μ *is an intercept, **M **is an incidence matrix whose columns are covariates for substitution, genotypic or haplotypic effects, **a **are effects to be estimated, *var*(**Ma**) = , **G **is a genomic relationship matrix [11-13], *ε *is the lack of fit, *var*(*ε*) = , hopefully small and will be **0 **if BV could be perfectly estimated as a linear function of observed marker genotypes. In different settings, **a **might be defined as a vector of fixed effects [14] or a vector of random effects [1]. Even when **a **is fixed, **Ma **is random because **M**, which contains genotypes, is random. However, in genomic analyses **M **is treated as fixed because the analysis is conditional on the observed genotypes. The philosophical issues related to the randomness of **M **and **a **are discussed in detail by Gianola [15] but for our context it is sufficient to define *var*(**Ma**) =  without explicitly specifying distributional properties of **M **or **a**.

Genotypes used as covariates in **Ma **are unlikely to capture all the variation in true genetic merit, either because they are not comprehensively covering the entire genome, or because linkage disequilibrium between markers and causal genes is not perfect. Knowledge of **E **is required in the analysis whether **a **is treated as a fixed (e.g. GLS) or random effect (e.g. BLUP). In practice with experiments that involve related animals, it is unreasonable to assume **E **has a simple form such as a diagonal matrix since that implies a zero covariance between lack of fit effects for different animals, however, it can be approximated using knowledge on the pedigree using the additive relationship matrix, **A **[16]. These lack of fit covariances can be accommodated by fitting a polygenic effect for each animal, in addition to the marker genotypes [17], or accounted for by explicitly modeling correlated residuals. For a non-inbred animal, , therefore  and the proportion of the genetic variance not accounted for by the markers can be defined to be . The scalar *c*, will be close to 0 if markers account for most of the genetic variation and close to 1 if markers perform poorly.

### A model using individual phenotypic records

In practice we do not have the luxury of using true BV as data in genomic prediction. A more common circumstance might involve training based on phenotypic observations that include fixed effects on phenotype denoted **Xb **where **X **is an incidence matrix for fixed non-genetic effects in **b**. An appropriate model equation for phenotypes is(2)

where **e **is a vector of random non-genetic or residual effects. In comparison to (1), the use of **y **for training involves the addition of the vectors **Xb **and **e **to the left- and right-hand side, inflating the variance and giving(3)

with  since cov(*ε*, **e'**) = **0**. This model can be fitted by explicitly including a random polygenic effect for *ε*, or by accounting for the non-diagonal variance-covariance structure of the residuals defined as var (*ε *+ **e**). Including a polygenic term is not typically done in genomic prediction analyses [12,18], and when undertaken does not seem to markedly alter the accuracy of genomic predictions [Habier D. Personal communication]. Assuming var (*ε *+ **e**) is a scaled identity matrix facilitates the computing involved in fitting this model, as the relevant mixed model equations can be modified by multiplying the left- and right-hand sides by the unknown scale parameter as is typically done in single trait analyses. However, this is not an option if residuals are heterogeneous, for example, because they involve varying numbers of repeated observations.

### A model using repeated records on the individual

Consider the circumstance where the training observations are a vector  representing observations that are the mean of *n *observations on the individual with *n *potentially varying. In that case, equation (3) becomes(4)

With , a diagonal matrix with elements  with  being the phenotypic variance, heritability *h*^2^, and repeatability *t*. Ignoring off-diagonal elements of **E**, the elements of the inverse of **R **with **R **= var(*ε*) + **D **would for non-inbred animals be . In fixed effects models, this matrix can be arbitrarily scaled for convenience. In univariate random effects models, a common practice is to formulate mixed model equations using the ratios of residual variance to variances of the random effects. Here, it makes sense to factor out the residual variance of one phenotypic observation, i.e. , from the expression for the residual variance of the mean of *n *observations. In this circumstance, a scaled inverse of the residual variance being  or equivalently(5)

which can be used for weighted regression analyses treating marker effects as fixed or random. When *c *= 0, the genetic effects can be perfectly explained by the model, and for *n *= 1, a single observation on the individual, the weight is 1 for any heritability. Scaling the weights is convenient because records with high information exceed 1 and the weights are trait independent which is useful when analysing multiple traits with identical heritability and information content.

### Offspring averages as data

In some cases the training data may represent the mean of *p *individual measurements on several offspring, rather than the mean phenotype of the genotyped animal. In that circumstance, the residual variance includes a genetic component for the mate and Mendelian sampling. For half-sib progeny means with unrelated mates and no common environmental variance, . However, the half-sib progeny mean contains only half the genetic merit of the parent, therefore the genotypic covariates need to be halved, or the mean doubled, in order to analyse data that includes records on genotyped individuals and records on offspring of genotyped individuals. The variance for twice the progeny mean is , and adding , factoring out  and inverting gives(6)

For full-sib progeny means the intraclass correlation of residuals will include a genetic component and perhaps a common environmental component (e.g. litter, with variance  and  giving  for unrelated parents. Adding variation due to  factoring out  and inverting gives(7)

This expression can be used as weights in the fixed or random regression of full-sib progeny means on parent average marker genotypes.

### Estimated breeding values as training data

An estimated breeding value, typically derived using BLUP, can be recognised as the true BV plus a prediction error. That is, . Accordingly, training on EBV might be viewed as extending the model equation in (1) by the addition of the prediction error, in the same way that (3) was derived by the addition of a residual nongenetic component. The model equation would therefore be(8)

There are at least two issues with this formulation of the problem, which may not be immediately apparent, and which both result from properties of BLUP. The first issue is that the addition of the prediction error term to the left- and right-hand side of (8) actually reduces rather than increases the variance, despite the fact that diagonal elements of  must exceed 0, in contrast to the addition of non-genetic random residual effects in (3). That is , whereas var(*g*_*i*_) < var(*y*_*i*_), due to shrinkage properties of BLUP estimators [19]. Generally,  but for BLUP  so that  implying . The reduction in variance of the training data comes about because prediction errors are negatively correlated with BV as can be readily shown since . This means that superior animals tend to be underevaluated (i.e. have negative prediction errors) whereas inferior animals tend to be overevaluated. This is a consequence of shrinkage estimation and prediction errors being uncorrelated with EBV, i.e. . In order to account for the covariance between the prediction errors and the BV, a model that accounted for such covariance would need to be fitted. Such models are computationally more demanding compared to models whereby the fitted effects and residuals are uncorrelated. The second issue resulting from the properties of BLUP, is that it is a shrinkage estimator, that shrinks observations towards the mean, the extent of shrinkage depending upon the amount of information. This is apparent if one considers the regression of phenotype on true genotype (i.e. BV) which is 1, whereas the regression of EBV on BV is equal to  ≤ 1, where  is the reliability of the EBV (for animal *i*) or squared correlation between BV and EBV. In the context of any marker locus, the contrast in EBV between genotypes at a particular locus is shrunk relative to the contrast that would be obtained if BV or phenotypes were used as data, with the shrinkage varying according to . We are, however, interested in estimating the effect of a marker on phenotype, but we get a lower value for the contrast if EBV with  ≤ 1 are used as data, rather than using phenotypes. A further complication is that training data based on EBV typically comprise individuals with varying . This problem can be avoided by deregressing or unshrinking the EBV.

### Deregressing estimated breeding values

The solution to the model fitting problems associated with the reduced variance of EBV and the inconsistent regression of EBV on genotype according to reliability can both be addressed by inflating the EBV. Rather than fitting (8), we will fit the linearly inflated data represented as **K** for some diagonal matrix **K**. That is, we will fit:(9)

for some matrix **K **chosen so that  and  is a constant. Since  then this expression will be 0 when . For this value *k*_*i*_, , a constant for all animals regardless of their reliability. Accordingly, the deregression matrix is **K **= *diagonal* and the deregressed observations are . Note in passing that the nature of the deregression will depend upon the EBV base. Genetic evaluations are typically adjusted to a common base before publication, by addition or subtraction of some constant. The EBV should be deregressed after removing the post-analysis base adjustment or by explicitly accounting for the base in the deregression procedure [20]. To show the dependence of the deregression to the post-analysis base, supposes that EBV are adjusted to a base, b. Then a linear contrast in deregressed EBV without removing the base effect is  unless . Marker effects are typically estimated as linear combinations of data, and will therefore be sensitive to the base adjustment.

A deregressed observation represents a single value that encapsulates all the information available on the individual and its relatives, as if it was a single observation with *h*^2 ^= *r*^2^. This can be shown by recognising that *h*^2 ^is the regression of genotype on phenotype. Taking the deregressed observation to be the phenotype, . Training on deregressed EBV is therefore like training on phenotypes with varying *h*^2^. Provided  > *h*^2^, training on deregressed EBV is equivalent to having a trait with higher heritability. However, as explained later, we recommend removing ancestral information from the deregressed EBV.

### Weighting deregressed information

Deregressed observations have heterogeneous variance when *r*^2 ^varies among individuals. The residual variance of a particular deregressed observation is  but  and  so the residual variance expression simplifies to . Ignoring the off-diagonal elements of var(*ε*) as before, the diagonals of the inverse of the residual variance after factoring out  are  which simplifies to give(10)

an expression analogous to (5) with *n *= 1 and *h*^2 ^= . Note that the weight in (10) approaches  as →1 in which case the weight tends to infinity as *c*→0. This is the same as would occur when the number of offspring *p*→∞, and *p *is used as a weight.

### Removing parent average effects

Animal model evaluations by BLUP using the inverse relationship matrix shrink individual and progeny information towards parent average (PA) EBV [21]. It makes sense to remove the PA effect as part of the deregression process for two reasons. First, some animals may have EBV with no individual or progeny information. These animals cannot usefully contribute to genomic prediction. This is apparent if one imagines a number of halfsibs with individual marker genotypes and deregressed PA EBV. These animals cannot add any information beyond what would be available from the common parent's genotype and EBV. Second, if any parents are segregating a major effect, about half the offspring will inherit the favourable allele and the others will inherit the unfavourable allele. However, the EBV of both kinds of offspring will be shrunk towards the parent average. Parent average effects can be eliminated by directly storing the individual and offspring deregressed information and corresponding *r*^2 ^during the iterative solution of equations carried out for the purposes of genetic evaluation [2]. In some cases researchers do not have access to the evaluation system used to create the EBV on their training populations. In those circumstances, it is necessary to approximate the evaluation equations and backsolve for deregressed information free of the effects of parent average. This can be done for one training animal at a time, given *h*^2 ^and knowledge of only the EBV (unadjusted for the base) and *r*^2 ^on the animal, its sire and its dam. First, compute parent average (PA) EBV and reliability for animal *i *with *sire *and *dam *as parents: , and . Assuming sire and dam are unrelated and not inbred, the additive genetic covariance matrix for PA and offspring is  with inverse . Using this result, recognise that the equations to be solved are:(11)

where  is information equivalent to a right-hand-side element pertaining to the individual,  and  reflects the unknown information content of the parent average and individual (plus information from any of its offspring and/or subsequent generations), *λ *= (1 - *h*^2^)/*h*^2 ^is assumed known. Define

then using the facts [19] that  and  leads to , and . Rearranging these equations, , and . The formula to derive the inverse of a 2 × 2 matrix applied to the coefficient matrix from (11) gives , and  for .

Equating these alternative expressions for *c*^*PA*, *PA *^leads to(12)

and equating the expressions for *c*^*i*, *i *^leads to(13)

Second, solve these nonlinear equations for  and . Although not obvious, there is a direct solution for  and . It can be derived by dividing (12) by (13), defining , and rearranging to get(14)

Substituting the expression for  in (14) into the denominator of (13), defining , and rearranging leads to a quadratic expression in , namely , which has a positive root that can rearranged to(15)

Application of (15) provides the solution for  that can be substituted in (14) to solve for , together enabling reconstruction of the coefficient matrix of (11).

Third, the right-hand side of (11) can be formed by multiplying the now known coefficient matrix by the known vector of EBV for PA and individual. The right-hand side on the individual, free of PA effects is  The equation to obtain an estimate of EBV for animal *i*, free of its parent average, , based only on , is  and the corresponding  for use in constructing the weights in (10) is given by . The deregressed information is , which simplifies to  and is analogous to an average. An iterative procedure using mixed model equations to simultaneously deregress all the sires in a pedigree, while jointly estimating the base adjustment and accounting for group effects was given by Jairath et al [20]. However, that method requires knowledge on the numbers of offspring of each sire.

### Double counting of information from descendants

Genetic evaluation of animal populations results in EBV that are a weighted function of the parent average EBV, any information on the individual, adjusted for fixed effects, and a weighted function of the EBV of offspring, adjusted for the merit of the mates [2]. The previous section has argued for the removal of parent average effects in constructing information for genomic analyses. It could be argued that information from genotyped descendants should also be removed to avoid double counting. This can be achieved during the evaluation process, and is desirable in the absence of selection. If the genotyped descendants are a selected subset, the removal of their information will lead to biased information on the individual. Simulation suggests that the double counting of descendants performance has negligible impact on genomic predictions (results not shown).

## Results

### Weights for different information sources

Comparative weights for individual and average of *n *individual observations using (5), and for progeny means of *p *halfsibs using (6) and deregressed EBV of varying reliability using (10) are in Table 1.

**Table 1:** Relative weights^*a *^for *n *phenotypic observations on the individual, *p *observations in twice the halfsib progeny mean with heritability 0.25 and repeatability 0.6, or deregressed EBV with reliability *r*^2 ^for varying values of *c*, the proportion of genetic variation for which genotypes cannot account

		*c*			
		
Information Source		0.8	0.5	0.25	0.1
Mean of *n *repeated records	*n*				
					
	1	0.79	0.86	0.92	0.97
	2	1.00	1.11	1.22	1.30
	5	1.19	1.35	1.52	1.65
	10	1.27	1.46	1.66	1.81

2 × mean of *p *half-sib offspring	*p*				
					
	5	0.79	0.86	0.92	0.97
	10	1.30	1.50	1.71	1.88
	20	1.94	2.40	3.00	3.53

Deregressed EBV with reliability *r*^2^	*r*^2^				
					
	0.1	0.31	0.32	0.32	0.33
	0.2	0.63	0.67	0.71	0.73
	0.3	0.96	1.06	1.16	1.23
	0.4	1.30	1.50	1.71	1.88
	0.5	1.67	2.00	2.40	2.73
	0.6	2.05	2.57	3.27	3.91
	0.7	2.44	3.23	4.42	5.68
	0.8	2.86	4.00	6.00	8.57
	0.9	3.29	4.91	8.31	14.21
	1.0	3.75	6.00	12.00	30.00

^*a*^Weights are diagonal elements of the inverse of the scaled residual variance-covariance matrix (with the scalar  factored out before inversion). Weights are relative to the information content of an individual observation with *c *= 0.

### Removing parent average effects

Suppose genomic training is to be undertaken for a trait using EBV available from national evaluations that have yet to be deregressed. Widely-used bulls have been genotyped and the EBV and *r*^2 ^of those bulls are available, along with corresponding information on the sire and dam of each bull. Such a trio might have values of  = 10,  = 0.97;  = 2,  = 0.36; and  = 15,  = 0.68. Given *h*^2 ^= 0.25, *λ *= 0.75/0.25 = 3, the PA information is , and . Using (15), with *α *= 5.97, *δ *= 0.523, then  = 9.16 which substituted in (14) gives  = 5.08.

Substituting these information contents into the coefficient matrix or left-hand side of (11) is  with inverse . These values correspond to  = 0.5 - 3 × 0.0558 = 0.33 and  = 1.0 - 3 × 0.1066 = 0.68 the reported  and  confirming the equations used to determine the information content. The right-hand side of (11) can then be reconstructed by multiplying the coefficient matrix by the vector of EBV as . The element of interest is the right-hand side element corresponding to the individual, obtained as  = -6 × 6 + 11.08 × 15 = 130. The deregressed information for use in subsequent analysis is obtained as  and the corresponding reliability of this information free of PA effects is  = 1.0 - 3/(5.08 + 3) = 0.63. The relevant scaled weight for use with the deregressed information on this individual assuming *c *= 0.5 can be found using (10) as . This implies that the deregressed information is 2.76 times more valuable than a single record on the individual.

## Discussion

The relative value of alternative information sources varies according to *c*, the parameter that reflects the ability of the genotypic covariates to predict genetic merit. Genomic prediction models that fit well have small values for *c *and result in greater relative emphasis of reliable information than is the case when the genomic prediction model fits poorly and the residual variation is dominated by contributions from lack-of-fit. For example, the mean of 20 halfsib progeny has about 3.6 times the value of the mean of 5 progeny when *c *is 0.1, and 2.5 times the value when *c *is 0.8. Deregressed EBV with reliability 1.0 are 11 times as valuable as reliability 0.5 when *c *is 0.1 but only 3 times as valuable when *c *is 0.5. These results indicate that collecting genotypes and phenotypes on training animals with low to moderate reliability will be of more relative value to genomic predictions that account for only 50% genetic variation (i.e. correlation 0.7 between genomic prediction and real merit) than they will for genomic predictions that account for a high proportion of variance.

The impact of the assumed *c *is to influence the relative value of individuals with reliable information, such as progeny test results, in comparison to individuals with information from less reliable sources, such as individual records. The use of too large a value of *c *will result in overemphasis of less accurate information in relation to more accurate information. The use of too small a value of *c *will result in too little emphasis on less accurate records. The correct value of *c *will not be known prior to training analyses but can be estimated from validation analyses. Training analyses could then be repeated using the estimated value of *c*. Alternatively, sensitivity to *c *could be assessed by training using a range of values. The sensitivity to *c *varies according to the heterogeneity of information content in the training data.

In practice, information sources of phenotypic data on training individuals can vary more widely than the examples derived in this paper. For example, training individuals might have their own and a mix of half-and fullsib progeny observed. In such cases, a practical approach is to first set up the mixed model equations that would be appropriate to estimate breeding values on the training individuals and use these to solve for the deregressed information [2]. This approach could also be useful in circumstances where training individuals do not all have the appropriate phenotypes. Consider a situation where some individuals have carcass measurements while others have correlated observations such as live animal ultrasound measures. A bivariate analysis of these two traits could be used to produce a single deregressed value for the carcass trait for each animal that accounted for appropriately weighted ultrasound information.

## Conclusions

The arguments put forward in this manuscript support the use of deregressed information, in agreement with practices adopted by many researchers [22]. The weighting factors proposed in this paper differ from any reported in the literature except when the parameter *c *= 0 in which cases the weights are effectively the same as those used by Georges et al. [5] and Spelman et al. [6]. In practice, the benefit of deregression and the subsequent weighting of alternative information sources will depend on the extent to which the number of repeat records, number of progeny and/or *r*^2 ^varies among individuals in the training population.

## Competing interests

The authors declare that they have no competing interests.

## Authors' contributions

DJG derived the formulae following debate with JFT and RLF as to appropriate weights for training analyses with disparate data. JFT derived the direct solution for removing parent average effects. DJG drafted the manuscript and RLF and JFT helped to revise and finalize it. All authors read and approved the final manuscript.
